# Distinct patterns of intratumoral immune cell infiltrates in patients with HPV-associated compared to non-virally induced head and neck squamous cell carcinoma 

**DOI:** 10.4161/21624011.2014.965570

**Published:** 2015-01-30

**Authors:** Simona Partlová, Jan Bouček, Kamila Kloudová, Eva Lukešová, Michal Zábrodský, Marek Grega, Jitka Fučíková, Iva Truxová, Ruth Tachezy, Radek Špíšek, Anna Fialová

**Affiliations:** 1 Sotio, Prague, Czech Republic; 2 Department of Immunology; 2nd Faculty of Medicine; Charles University; Motol University Hospital; Prague, Czech Republic; 3 Department of Otorhinolaryngology and Head and Neck Surgery; 1st Faculty of Medicine; Charles University and Motol University Hospital; Prague, Czech Republic; 4 Institute of Microbiology ASCR; Prague, Czech Republic; 5 Department of Experimental Virology; Institute of Hematology and Blood Transfusion; Prague, Czech Republic; 6 Department of Genetics and Microbiology; Faculty of Science; Charles University; Prague, Czech Republic; 7 Department of Pathology and Molecular Medicine; 2nd Faculty of Medicine; Charles University and Motol University Hospital; Prague, Czech Republic

**Keywords:** CD8^+^ T lymphocytes, HNSCC, HPV, PD-1, Tim-3, Cox-2, cyclooxygenase 2, HNSCC, head and neck squamous cell carcinoma, HPV, human papillomavirus, mDC, myeloid dendritic cell, PD-1, programmed cell death 1, pDC, plasmacytoic dendritic cell, PD-L1, programmed cell death-ligand 1, Tim-3, T cell immunoglobulin and mucin protein 3, Treg, regulatory T cell

## Abstract

Human papillomavirus (HPV) infection is one of the most important etiologic causes of oropharyngeal head and neck squamous cell carcinoma (HNSCC). Patients with HPV-positive HNSCC were reported to have a better clinical outcome than patients with HPV-negative cancers. However, little is known about the possible causes of different clinical outcomes. In this study, we analyzed a detailed immune profile of tumor samples from HNSCC patients with respect to their HPV status. We analyzed the characteristics of immune cell infiltrates, including the frequency and distribution of antigen-presenting cells and naïve, regulatory and effector T cells and the cytokine and chemokine levels in tumor tissue. There was a profound difference in the extent and characteristics of intratumoral immune cell infiltrates in HNSCC patients based on their HPV status. In contrast to HPV-negative tumor tissues, HPV-positive tumor samples showed significantly higher numbers of infiltrating IFNγ^+^ CD8^+^ T lymphocytes, IL-17^+^ CD8^+^ T lymphocytes, myeloid dendritic cells and proinflammatory chemokines. Furthermore, HPV-positive tumors had significantly lower expression of *Cox-2* mRNA and higher expression of PD1 mRNA compared to HPV-negative tumors. The presence of a high level of intratumoral immune cell infiltrates might play a crucial role in the significantly better response of HPV-positive patients to standard therapy and their favorable clinical outcome. Furthermore, characterization of the HNSCC immune profile might be a valuable prognostic tool in addition to HPV status and might help identify novel targets for therapeutic strategies, including cancer immunotherapy.

## Introduction

HNSCC is a heterogeneous group of tumors located in the oral cavity, oropharynx, hypopharynx and larynx. Originally, tobacco and/or alcohol exposure were the main risk factors for HNSCC, but in an expanding subset of patients with oropharyngeal carcinoma, HPV infection has been described in the last two decades as a crucial etiologic agent.^1,2^ Although patients with HPV-associated tumors are more often diagnosed at advanced stages of the disease with large metastatic lymph nodes, their prognosis is reported to be significantly better than that of patients with non-HPV induced cancers.^3,4^

Despite the improved response of HPV-positive HNSCC to conventional treatment involving a combination of surgery, radiation therapy and chemotherapy, HPV-positive cell lines were shown to be more resistant to radiation and cisplatin *in vitro* when compared to HPV-negative cells. However, *in vivo*, HPV-positive tumors were more sensitive to radio- and chemotherapy in immunocompetent mice. Importantly, neither radiotherapy nor cisplatin therapy cured immunocompromised mice, indicating an important role for the immune system in HPV-positive HNSCC outcome.^5^ Although contradictory results recently published by Kimple et al.^6^ showed enhanced radiation sensitivity in HPV-positive cancer cell lines, this finding does not explain why HPV-positive patients treated with surgery alone also have a better prognosis.^3^ Moreover, in addition to the high proportion of relapses, especially in HPV-negative patients, conventional therapy remains associated with significant toxicity. Therefore, there is great interest in developing less toxic and more targeted therapies, including immunotherapy. Consequently, a better understanding of the interplay between the tumor microenvironment, HPV and the infiltrating immune cells is essential.

Indeed, characterization of the adaptive immune response has been shown to be an important prognostic tool in a wide range of carcinomas, potentially even more relevant than the current cancer staging system.^7-11^ In HNSCC, recently published studies indicate that the assessment of the level of circulating CD8^+^ T lymphocytes,^12^ the extent of tumor infiltration by CD8^+^ T lymphocytes and the ratio of infiltrating CD8^+^/FoxP3^+^ T lymphocytes^13,14^ might have a prognostic significance. However, a complex profile of the particular tumor-infiltrating immune cell subsets, including antigen-presenting cells, has not been evaluated to date.

In this study, we analyzed the distribution and phenotype of CD8^+^ and CD4^+^ T cell subsets, dendritic cell subsets (DCs) and monocytes/macrophages as well as the chemokine and cytokine profile in fresh HNSCC samples with regard to HPV status. Our findings confirm that HPV-positive tumor samples show a distinct immunologic profile compared to HPV-negative samples, with high levels of infiltrating IFNγ^+^ CD8+ T lymphocytes, IL-17^+^ CD8^+^ T lymphocytes (Tc17 lymphocytes), myeloid DCs and spontaneously produced proinflammatory chemokines and cytokines. Additionally, HPV-positive samples expressed significantly lower levels of *Cox-2* mRNA and higher levels of *PD-1* mRNA than HPV-negative samples.

## Results

### HPV-positive tumors are mostly localized in the oropharynx

As expected, only the HPV 16 type was detected in all of the samples that were positive for HPV DNA. The expression of HPV 16 E6 mRNA was detected in 45.5% (*n* = 20) of patients; 55.0% (*n* = 11) of the HPV-positive tumors were localized in the tonsils, 40.0% (*n* = 8) at the base of the tongue and 5.0% (*n* = 1) at the base of the oral cavity (**Table 1**). Lymph node metastases were histologically confirmed (pN^+^) in 90.5% of HPV-positive and 66.7% of HPV-negative patients, but this difference did not reach a statistical significance. The tumor grade and the stage were equivalent in patients with HPV-positive and HPV-negative HNSCC.

**Table 1.** Frequency of HPV-positive and HPV-negative tumors

	HPV negative -		HPV positive +	
	N	%	N	%
Total	24	54.5	20	45.5
Tongue	9	37.5	8	40.0
Tonsil	2	8.3	11	55.0
Larynx	8	33.3	0	0
Others	5	20.8	1	5.0

### Patients with HPV-positive HNSCC have significantly higher levels of tumor-infiltrating CD8^+^ T cells with the capacity to produce IFNγ and IL-17 after *in vitro* stimulation.

**Figure 1.**
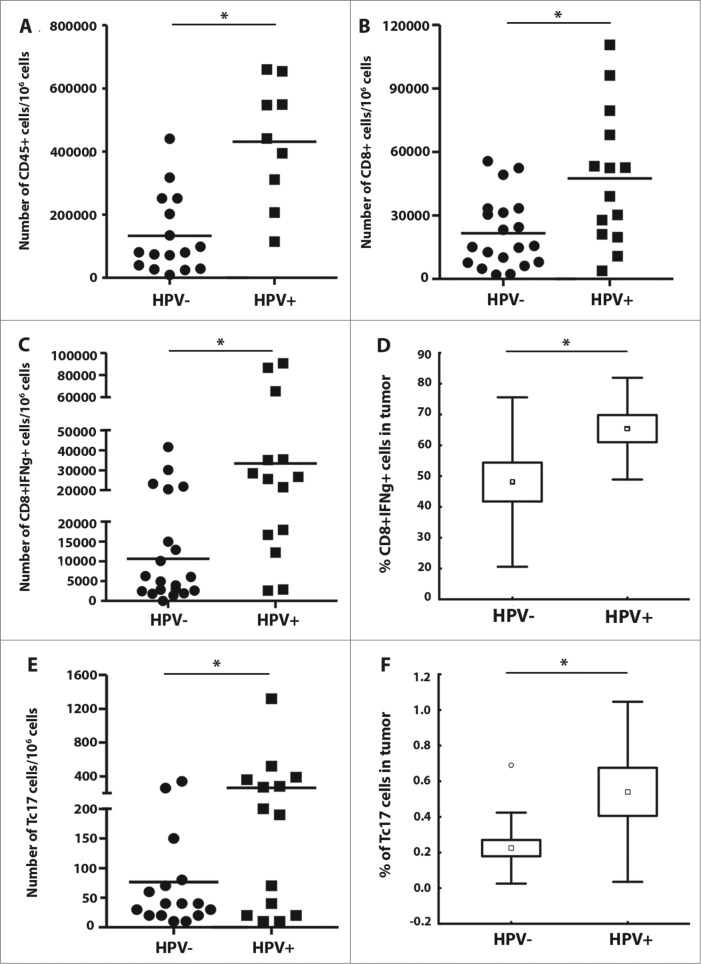
Proportions of leukocytes within tumor tissues of head and neck squamous cell carcinoma (HNSCC) patients (*n* = 44) according to human papillomavirus (HPV)-status. To evaluate the pattern of the immune cell infiltrates, tumor-derived single cell suspensions were stimulated with PMA and ionomycin in the presence of Brefeldin A and analyzed by flow cytometry. (**A–C, and E**) The data are expressed as the numbers of CD45^+^ cells, CD8^+^ cells, CD8^+^IFNγ^+^ cells and Tc17 in 1 × 10^6^ isolated tumor-derived cells; the line represents the mean value. (**D and F**) Box plots represent the proportions of CD8^+^IFNγ^+^ cells and CD8^+^IL-17^+^ (Tc17 cells) among the tumor-infiltrating CD8^+^ T cells. The boundaries of the box indicate the standard error of the mean (SEM), and the lines in the box represent the median. Whiskers indicate the standard deviation (SD). * *p* < 0.05 (General Linear Models; age was added as a covariate).

The presence of tumor-infiltrating leukocytes, particularly CD8^+^ T cells, was shown to be a strong prognostic marker in various types of cancer; therefore, we analyzed the numbers and proportions of tumor-infiltrating immune cells in HPV-positive and HPV-negative HNSCC patients. The levels of tumor-infiltrating CD45^+^ leucocytes were significantly higher in HPV-positive tumor samples (**Fig. 1A**). As expected, there were significantly higher numbers of CD8^+^ T cells in HPV-positive tumor tissue samples compared to HPV-negative samples (**Fig. 1B**). Additionally, significantly higher proportions of CD8^+^ cells from HPV-positive samples produced IFNγ (**Fig. 1C and D**) or IL-17 (**Fig. 1E and F**) upon PMA and ionomycin stimulation *in vitro*.

### Patients with HPV-positive HNSCC have higher numbers of CD4^+^ T cells in tumor tissue

**Figure 2.**
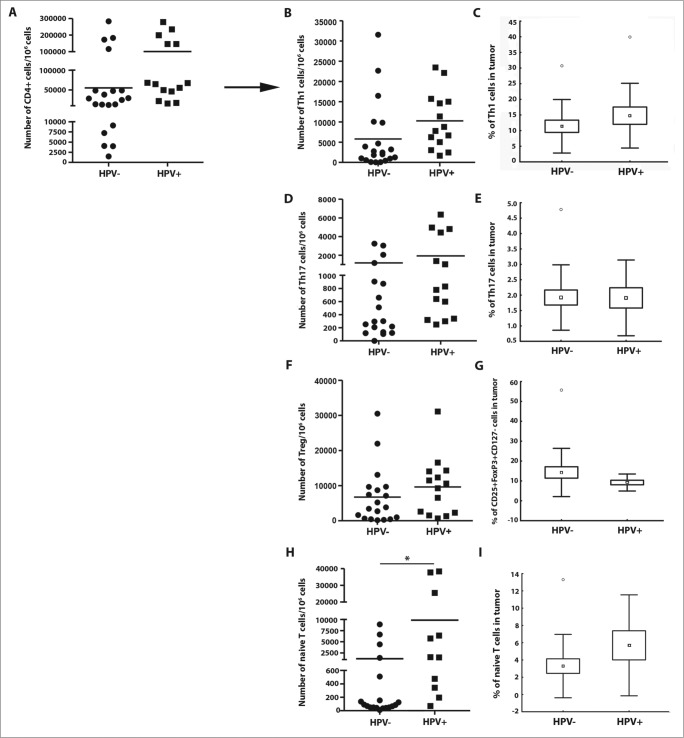
The frequency of CD4^+^ T cell subsets in the tumor tissues (*n* = 44) with regard to human papillomavirus (HPV)-status. To evaluate the subtypes of tumor-infiltrating CD4^+^ T cells, tumor-derived single cell suspensions were stimulated with PMA and ionomycin in the presence of Brefeldin A and analyzed by flow cytometry. (**A, B, D, F, and H**) The plots represent the numbers of Th1 cells, Th17 cells, Tregs or naïve T cells within 10^6^ isolated tumor-derived cells; the lines in the box represent the median. (**C, E, G, and I**) The data are expressed as the proportion of Th1 cells, Th17 cells, Tregs and naïve T cells, respectively, among the tumor-infiltrating CD4^+^ cells. The boundaries of the box indicate the SEM, and the lines in the box represent the mean. Whiskers indicate the SD * *p* < 0.05 (General Linear Models; age was added as a covariate).

We observed a trend to an increase in the numbers of total CD4^+^ T cells as well as IFNγ-producing CD4^+^ cells (Th1 cells) in the HPV-positive tumor samples (*p* < 0.1) (**Fig. 2A–C**). The proportion of Th17 cells did not show any differences between HPV-positive and -negative tumors (**Fig. 2D and E**); however, single cell suspensions isolated from HPV-positive tumors produced significantly higher levels of IL-17 upon PMA and ionomycin stimulation *in vitro* (p = 0.030) (**Fig. 4C**). Additionally, we observed a slightly lower proportion of Tregs in HPV-positive tumors (**Fig. 2G**). None of the subsets of CD4^+^ T cells listed above showed any statistically significant differences between patients with HPV-positive and -negative tumors, but significantly higher numbers of naïve T cells were detected in HPV-positive tumor tissues compared to HPV-negative tumor samples (*p* = 0.018) (**Fig. 2H and I**).

**Figure 3.**
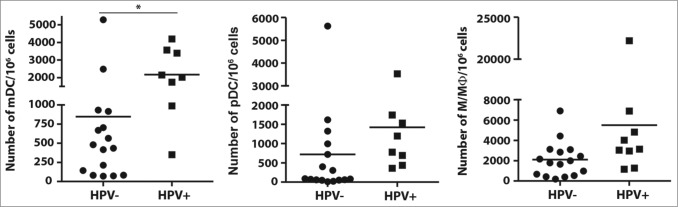
Numbers of dendritic cells (DCs) and monocytes/macrophages within the tumor tissue of head and neck squamous cell carcinoma (HNSCC) patients (*n* = 26) in relation to human papillomavirus (HPV)-status. To assess the pattern of tumor-infiltrating antigen presenting cells, fresh tumor-derived single cell suspensions were analyzed by flow cytometry. (**A**) The data are expressed as the numbers of myeloid dendritic cells (mDCs, characterized as CD45+LIN-HLA-DR+CD14-CD11c+), plasmacytoid dendritic cells (pDCs, characterized as CD45+LIN-HLA-DR+CD14-CD123+) and monocytes/macrophages (Mo/Mᶲ, characterized as CD45+LIN-HLA-DR+CD14+) in 10^6^ isolated tumor-derived cells. The lines represent the mean value. * *p* < 0.05 (General Linear Models; age was added as a covariate).

**Figure 4.**
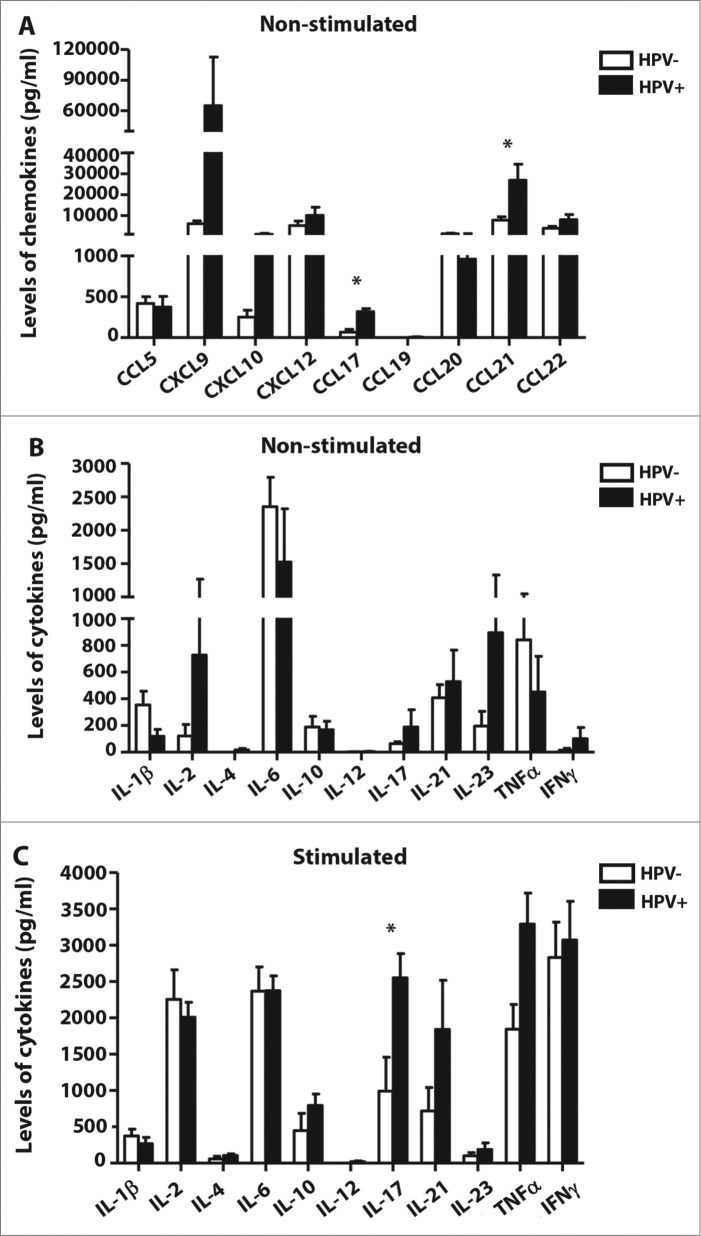
Chemokine and cytokine profiles of tumor-derived single cell suspensions (HPV-negative samples: *n* = 7; HPV-positive samples: *n* = 7). Supernatants of tumor-derived single cell suspension cultures were analyzed using a Quantibody Array Kit (Raybiotech, Norcross, GA). (**A**) The white columns represent the mean spontaneous chemokine production after 24 h for culture supernatants from HPV-negative patients; the black columns represent the mean production from human papillomavirus (HPV)-positive patients. (**B**) The white columns represent the mean spontaneous cytokine production after 24 h for culture supernatants from HPV-negative patients; the black columns represent the mean production from HPV-positive patients. (**C**) The white columns represent the mean cytokine production upon PMA and ionomycin stimulation after 24 h for culture supernatants from HPV-negative patients; the black columns represent the mean production from HPV-positive patients. All of the error bars indicate the SEM * *p* < 0.05 (General Linear Models; age was added as a covariate).

### HPV-positive HNSCC samples have increased numbers of tumor-infiltrating antigen presenting cells

Additionally, we also analyzed subsets of antigen presenting cells, namely mDCs, pDCs and monocytes/macrophages in the tumor tissue. We observed an increased frequency of all of these cell populations in patients with HPV-positive tumors. The total numbers of mDCs in HPV-positive tumor samples showed a statistically significant increase compared to HPV-negative samples; however, the frequency of pDCs and monocytes/macrophages were not statistically significantly different (**Fig. 3**). As expected, we detected high proportions of CD16^+^ HNSCC-infiltrating mDCs, but these proportions did not differ between HPV-negative and HPV-positive tumor tissues (70.9 ± 4.5% and 65.5 ± 2.3%, respectively).

### HPV-positive tumor tissue-derived single cell suspensions produced higher levels of chemokines, but the cytokine profile was not significantly different

To evaluate whether the increased frequency of leukocytes in HPV-positive tumor samples could be caused by the recruitment of leukocytes to tumor sites rather than their polarization *in situ*, as shown in our previous study,^15^ we analyzed the chemokine and cytokine profiles of the tumor-derived cell culture supernatants.

In the supernatants from unstimulated cell cultures, high levels of CXCL9, CXCL10, CXCL12, CCL20, CCL21 and CCL22 were detected. Of the other chemokines measured, only CCL5, CCL17 and CCL19 were at low levels (**Fig. 4A**). Markedly higher production of CXCL9, CXCL10, CXCL12, CCL17 and CCL21 was detected in the supernatants of HPV-positive patient samples compared to HPV-negative patient samples; however, only the levels of CCL17 and CCL21 showed statistically significant differences (*p* = 0.023 and *p* = 0.040, respectively). As expected, the levels of CXCL12 positively correlated with the lymph node status (average values: N0 = 0 pg/mL and N1–3 = 8331.3 ± 2357.1 pg/mL).

Surprisingly, even in unstimulated cell cultures, we were able to detect most of the cytokines analyzed with the exception of IL-4 and IL-12 (**Fig. 4B**). HPV-positive cell cultures tended to produce higher levels of IL-2, IL-17, IL-23 and IFNγ and lower levels of IL-1 β, but these differences were not statistically significant. In supernatants of HPV-positive tumors upon PMA and ionomycin stimulation we detected higher levels of IL-10, IL-17, IL-21, TNFα and IFNγ compared to supernatants of HPV-negative tumor samples; however, only the levels of IL-17 showed statistically significant differences (*p* = 0.030) (**Fig. 4C**).

### HPV-positive tumor samples expressed lower levels of *Cox-2* and Tim-3 mRNA and higher levels of PD-1 mRNA

In addition to the flow cytometry data described above, we analyzed the mRNA expression levels of four regulatory genes, *Cox-2*, *PD-1*, *PD-L1* and *Tim-3*, in the tumor tissue samples and in metastatic and control lymph nodes of patients with HPV-positive and HPV-negative tumors.

**Figure 5.**
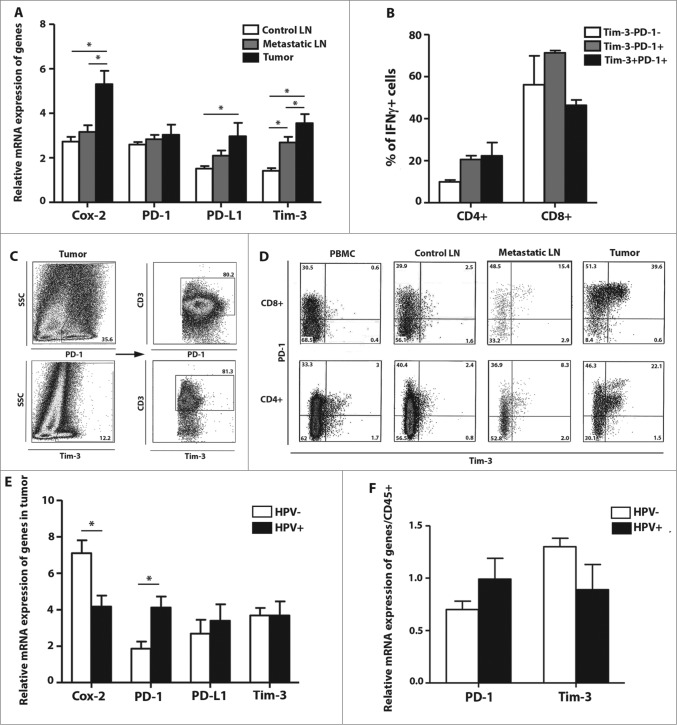
The levels of cyclooxygenase 2 (*Cox-2*), programmed cell death 1 (PD-1), programmed cell death-ligand 1 (PD-L1) and T cell immunoglobulin and mucin domain containing protein-3 (Tim-3) in control and metastatic lymph nodes and tumor tissue from head and neck squamous cell carcinoma (HNSCC) patients. (**A**) The white columns represent the relative mRNA expression of *Cox-2*, *PD-1*, *PD-L1* and *Tim-3* in control lymph nodes (LN; *n* = 14); the gray columns represent the relative mRNA expression of genes in metastatic lymph nodes (*n* = 14); the black columns represent the relative mRNA expression of these genes in tumor tissues (*n* = 14). To assess the expression levels of mRNA, cDNA was synthesized from total tumor and lymph node RNA and amplified by quantitative real time PCR. As an internal reference, β-actin housekeeping gene was used. (**B**) The columns represent the mean proportion of IFNγ^+^ cells among Tim-3-PD-1- (white column), Tim-3-PD-1+ (gray column) and Tim-3+PD-1+ (black column) cells in the tumor tissue (*n* = 6). To analyze the IFNγ production, tumor-derived single cell suspensions were stimulated with PMA and ionomycin in the presence of Brefeldin A and analyzed by flow cytometry. (**C**) Dot plots show the expression of CD3^+^ on PD-1^+^ or Tim-3^+^ tumor-infiltrating cells from a representative patient. (**D**) Dot plots are gated on CD3^+^CD8^+^ cells (upper line) and CD3^+^CD4^+^ cells (lower line) and show the expression of *Tim-3* and *PD-1* in the peripheral blood, control LN, metastatic LN and tumor tissue from a representative patient. The columns represent the mean relative mRNA expression of *Cox-2*, *PD-1*, *PD-L1* and *Tim-3* or *PD-1* and *Tim-3*, respectively, in HPV- (white columns) and HPV+ (black columns) tumor samples (*n* = 14) with (**F**) or without (**E**) normalization to the expression level of CD45^+^ mRNA. All of the error bars indicate the SEM * *p* < 0.05 (**A**, paired t-test; **B**, Friedman ANOVA; **E and F**, General Linear Models; age was added as a covariate).

We observed markedly higher expression of all of the analyzed genes except *PD-1* in tumor tissues and metastatic lymph nodes compared to control lymph nodes, regardless of HPV status (**Fig. 5A**). In comparison to the HPV-positive tumor tissues, we detected a significant increase in the expression of the negative prognostic marker *Cox-2* in the HPV-negative samples (p = 0.016). On the contrary, we observed a significantly higher expression of PD-1 in HPV-positive tumor tissues compared to HPV-negative samples (*p* = 0.018). The mRNA expression level of Tim-3 was similar in both groups of patients (**Fig. 5E**). However, because we observed markedly higher numbers of CD45^+^ cells in the HPV-positive tumor tissues (**Fig. 1A**), we decided to normalize the expression levels of PD-1 and Tim-3 mRNA to the expression level of CD45^+^ mRNA. When the results were normalized to CD45^+^ mRNA expression, we observed higher levels of PD-1 and lower levels of Tim-3 in HPV-positive tissue samples compared to HPV-negative tumors, but these differences were not statistically significant (**Fig. 5F**).

In addition to mRNA expression, we analyzed the expression of *PD-1* and *Tim-3* on freshly isolated tumor-derived cells using flow cytometry. Both *PD-1* and *Tim-3* were mainly expressed on CD3^+^ T lymphocytes (**Fig. 5C**). Whereas *PD-1* was frequently expressed on CD8^+^ and CD4^+^ T cells in all of the tissues analyzed, particularly in the peripheral blood, control lymph nodes, metastatic lymph nodes and tumor tissue, *Tim-3* was mainly expressed on tumor-infiltrating T-lymphocytes (**Fig. 5D**). As expected, we observed substantially decreased production of IFNγ by Tim-3+PD-1+ CD8^+^ T cells compared to both Tim-3-PD-1-CD8^+^ T cells and Tim-3-PD-1+ CD8^+^ T cells (**Fig. 5B**), whereas there was no difference in IFNγ production in CD4^+^ T cells with regard to the expression of PD-1 and Tim-3.

## Discussion

There are two major causative agents of HNSCC, namely tobacco and/or alcohol consumption and a high-risk HPV infection. HPV-associated tumors are known to show a different molecular profile than tobacco/alcohol-induced cancers, similar to HPV-positive cervical tumors. Although HPV-positive HNSCC patients are often diagnosed at a late stage of the disease with developed nodal metastases, their prognosis is significantly better compared to HPV-negative cancers.^2^ It has been suggested that the improved response of HPV-positive HNSCC patients to the conventional treatment was related to the immune system^5^; however, a detailed analysis of the pattern of tumor-infiltrating immune cells in HPV-positive compared to HPV-negative HNSCC tissues has not been performed to date.

In this prospective study, we analyzed the immune profile of 54 fresh HNSCC samples with regard to their HPV status. We report that HPV-positive tumors have a markedly different immunologic profile compared to HPV-negative tumors, with high levels of infiltrating CD8^+^ IFNγ^+^ T lymphocytes, Tc17 lymphocytes, naïve CD4^+^ T lymphocytes and myeloid DCs. HPV-positive tumor tissue-derived cell cultures produced markedly higher levels of chemokines, namely CXCL9, CXCL10, CXCL12, CCL17 and CCL21, and slightly higher levels of the cytokines IL-2, IL-17, IL-23 and IFNγ. Additionally, HPV-positive samples expressed significantly lower levels of *Cox-2* mRNA and higher levels of PD-1 mRNA.

It has been convincingly shown that a high density of tumor-infiltrating CD8^+^ T lymphocytes is predictive of a favorable clinical outcome in different types of cancers, including HNSCC.^7,9,10,14,16^ Because most of the studies focusing on the quantification of tumor-infiltrating immune cells in HNSCC were retrospective and based on immunohistochemical data, the detailed phenotype of CD8^+^ T cells had not yet been evaluated. Importantly, recent HNSCC studies^17,18^ suggest that other characteristics of the intratumoral CD8^+^ T lymphocytes, such as their location within the tumor, PD-1 expression, as well as the expression of HLA-class I, SCINDERIN and EPHRIN-A1 in the tumor cells might have a strong impact on the prognostic value of the CD8^+^ T cells. Therefore, not only a quantitative, but also a qualitative analysis of the immune cell infiltrates seems to be crucial for the identification of clinically relevant prognostic markers. Our data show that tumor-infiltrating CD8^+^ T cells in HPV-positive tumor samples not only were more frequent but also had a higher capacity to produce IFNγ and IL-17 upon PMA and ionomycin stimulation compared to HPV-negative tumors, indicating a stronger immune response.

The role of CD4^+^ T cells in anticancer immunity is more controversial. Most of the studies examining tumor-infiltrating CD4^+^ T cells as possible prognostic markers focused on regulatory T cells. The role of Tregs seems to differ according to the type and etiology of the cancer. On one hand, Tregs are known to be the key mediators of tumor immune suppression, and high numbers of tumor-infiltrating Tregs have been related to poor outcome in many types of cancer.^19-22^ On the other hand, Tregs have been described as a positive prognostic factor in colorectal cancer and lymphomas.^23,24^ In HNSCC, Badoual et al.^25^ showed that high numbers of tumor-infiltrating Tregs positively correlated with locoregional control. Similarly, other groups studying these cells confirmed the positive correlation between the numbers of intratumoral Tregs and the overall survival.^26,27^On the contrary, Nasman et al.^13^ demonstrated that a high CD8^+^/FoxP3^+^ T cell ratio correlated with longer disease-free survival. With regard to the significance of peripheral blood Tregs, Boucek et al.^28^ showed that high levels of Tregs in the peripheral blood of HNSCC patients at the time of primary diagnosis might predict an early recurrence of the disease. In our study, we found higher numbers of CD4^+^ T cells in HPV-positive tumor samples, with slightly higher proportions of Th1 cells and a significantly higher number and frequency of naïve T cells. No statistically significant differences were observed in the numbers and proportions of Tregs and Th17 cells. For understanding the possible relationship between the increased immune infiltration of HPV-positive tumors and HPV-specific immune response, it will be important to analyze the specificity of infiltrating T cells. It is conceivable that, at least some, infiltrating T cells in HPV-positive head and neck tumors will be HPV specific as already suggested by recent studies.^29^ These results are comparable to what is found in anogenital HPV16 induced lesions.^30,31^ However, the detailed analysis of tumor specificity and viral specificity of T cells present in the head and neck tumors needs to be performed in future studies.

In addition to the increased lymphocytic infiltration, we observed higher numbers of mDCs and slightly higher numbers of pDCs and monocytes/macrophages in HPV-positive tumors. High numbers of CD68^+^ macrophages infiltrating HNSCC were shown to correlate with lymph node metastasis, extracapsular spread and an advanced stage of disease.^32^ In accordance with these results, we found that the numbers of monocytes/macrophages significantly positively correlated with the lymph node status (p = 0.048, data not shown). The prognostic impact of DC subtypes on the tumor microenvironment is less clear. Most likely, the phenotype, not only the number, of DCs might be crucial.^10^ It was shown that in HNSCC, monocytes and DCs express the low-affinity FcγRIII (CD16).^33^ As HNSCC patients are known to have high levels of antigen-antibody immune complexes^34^ that activate monocytes and DCs via CD16,^35^ CD16 crosslinking might promote pro-tumor and angiogenic activities.^36^ Indeed, in our study, we also found a markedly higher number of tumor-infiltrating CD16+ mDCs (70.9 ± 4.5% for HPV-negative tumors and 65.5 ± 2.3% for HPV-positive tumors) than CD16-mDCs. However, the prognostic value of these mDC subtypes remains to be elucidated.

In addition to differences in immune cell infiltrates, we also observed a markedly different chemokine profile in HPV-positive and HPV-negative tumor tissue-derived cell cultures. HPV-positive tumor tissue-derived cell cultures produced much higher levels of chemokines, namely CXCL9, CXCL10, CXCL12, CCL17 and CCL21. The chemokine CXCL12 (SDF-1) and its receptor CXCR4 were shown to play a key role in regulating the trafficking of cancer cells to sites of metastases.^37,38^ Indeed, in our study, only cell cultures derived from patients with lymph node metastases produced CXCL12 (N0 = 0 pg/mL compared to N1–3 = 8331.3 ± 2357.1 pg/mL). As nodal metastases were detected in 90.5% of the patients with HPV-positive and 66.7% of the patients with HPV-negative tumor samples, the differences in CXCL12 levels most likely reflect this fact. The levels of CCL17 (TARC), a ligand for CCR4, positively correlated with the numbers of tumor-infiltrating Th17, Th1 and CD8^+^ T lymphocytes. In mice, CCL17 was shown to be mainly produced by mature DCs of myeloid origin.^39^ We did not observe any correlation between CCL17 production and the numbers of mDCs in the tumor tissue; however, we observed a significant positive correlation between the CCL17 levels and the numbers of tumor-infiltrating pDCs (r = 0.91; *p* < 0.001). Surprisingly, the levels of CCL21, a ligand for CCR7, positively correlated only with the frequency of Th17 cells but not with the number or frequency of DCs and naïve T cells. Although tumor-derived cells of HPV-positive tumors also expressed higher levels of proinflammatory cytokines, namely IL-2, IL-17, IL-23 and IFNγ, these differences did not reach statistical significance due to high variability in the samples.

Analysis of the mRNA expression levels of four regulatory genes, *Cox-2*, *PD-1*, *PD-L1* and *Tim-3*, also showed differences associated with HPV status. The expression of *Cox-2*, which specifically catalyzes the production of prostaglandins, is undetectable in most healthy tissues but is usually overexpressed in inflammation, premalignant lesions and tumors. Functionally, *Cox-2*-derived prostaglandins were shown to promote angiogenesis and induce tumor invasion.^40,41^ Consequently, high *Cox-2* expression was associated with an unfavorable outcome in breast cancer patients.^42^ We detected basal expression of *Cox-2* in control lymph nodes and significant overexpression of *Cox-2* in tumor tissues from our cohort of HNSCC patients (**Fig. 5A**). The levels of *Cox-2* mRNA negatively correlated with the numbers of tumor-infiltrating Th1 and Th17 lymphocytes (*p* = 0.002 and *p* = 0.003, respectively, data not shown) and positively correlated with the mRNA expression of *Tim-3* (*p* = 0.008, data not shown). Interestingly, the HPV-positive tumor samples expressed significantly lower levels of *Cox-2* mRNA compared to the HPV-negative samples, which is in accordance with a previous report stating an improved outcome in HPV-positive HNSCC patients.^2^

The surface receptors *PD-1* and *Tim-3* belong to a group of immune checkpoint proteins that decrease T-cell reactivity and were identified, together with CTLA-4 and Lag-3, as benchmarks for exhausted T cells.^43^ Dysfunctional, exhausted T cells develop after long-term exposure to a high antigen load^44^ and are incapable of exhibiting robust effector responses to further antigen re-challenge.^45,46^ Interestingly, in cancer, dysfunctional T cells expressing *Tim-3* and *PD-1* were only found in tumor tissue but not in the peripheral blood.^47^ Importantly, tumor-infiltrating CD8^+^ Tim-3+ PD-1+ cells exhibited a surface phenotype that is consistent with effector/memory T cells, indicating that exhausted T cells emerge from effector T cells.^48^ Indeed, it was recently shown that exhausted T cells are successfully re-functionalized by blocking checkpoint receptors. Consequently, cancer immunotherapy using T-cell checkpoint inhibitors has become one of the most promising new therapeutic approaches.

In agreement with published data, we only found high proportions of Tim-3+ PD-1+ T cells in tumor tissue but not in the peripheral blood and control lymph nodes of HNSCC patients. On the contrary, Tim-3-PD-1+ T cells were observed in all of the tissues analyzed. Consistent with the flow cytometry data, we detected significantly higher levels of Tim-3 but not *PD-1* mRNA in tumor tissue compared to control lymph nodes. To examine the capacity of Tim-3-PD-1-, Tim-3-PD-1+ and Tim-3+PD-1+ tumor-infiltrating T cells to produce IFNγ, we analyzed the phenotype of these cells after *in vitro* stimulation with PMA and ionomycin using flow cytometry. Although we detected IFNγ-producing cells in all of the subtypes of T cells tested, the proportions of IFNγ^+^ T cells were markedly lower in CD8^+^PD-1+Tim-3+ T cells compared to CD8^+^PD-1+Tim-3- and CD8^+^PD-1-Tim-3- T cells, as expected. These data indicate that Tim-3 together with PD-1 might be considered a better exhaustion marker in HNSCC-infiltrating CD8^+^ T cells than PD-1 alone.

Badoual et al.^18^ showed that high levels of tumor-infiltrating PD-1+ T cells correlated with better survival in HNSCC patients. In agreement with these results, we observed higher *PD-1* expression in HPV-positive tumor samples compared to HPV-negative tumors. In a preclinical model, Badoual et al.^18^ showed that partial grafting of the HPV E7-expressing TC-1 epithelial cell line, which constitutively expresses PD-L1, is dependent on the presence of HPV-specific PD-1+ CD8^+^ T cells. An anti-PD-L1 monoclonal antibody vaccine further enhanced this immune response. Here, we confirmed that *PD-L1* was markedly more expressed in tumor tissue than in the control lymph nodes. As *Tim-3* expression was also tumor tissue specific, these two molecules, instead of *PD-1* alone, might be a very promising target for immunotherapy in HNSCC.

Taken together, our data show that HPV-positive tumor tissues have a distinct immune profile compared to HPV- negative tumors. Substantial infiltrates of immune cells are usually associated with a good prognosis and indicate a strong past antitumor immune response in HPV-positive tumors, which might be reactivated/reprogrammed by not only a targeted immunotherapy approach, but even during the standard therapy. Better understanding of targets of the immune response in HPV-positive vs. HPV-negative tumors and of mechanisms directing the recruitment of immune cells to the tumor will hopefully lead to the design of successful immunotherapeutic strategies.

## Materials and Methods

### Patients and samples

Blood samples, primary HNSCC specimens metastatic and control lymph nodes were obtained from 54 patients immediately after radical surgery at the Department of Otorhinolaryngology and Head and Neck Surgery, 1st Faculty of Medicine, Charles University and Motol University Hospital in Prague between April 2011 and November 2013. The patients enrolled in this study had not received any neoadjuvant chemo- or radiotherapy. All of the patients signed an informed consent approved by the Institutional Review Board of the University Motol. The clinical-pathological characteristics of the patients are summarized in **Table 2**.

**Table 2.** Clinical-pathological characteristics of the patients

Variable	No.	%
Total no. of patients	54	
Age		
Mean	62	
Range	38–78	
Sex		
Male	44	81.5
Female	10	18.5
Tumor grade		
1	8	14.8
2	30	55.6
3	16	29.6
4	0	0
Nodal status		
N0	13	24.1
N1	11	20.4
N2	28	51.9
N3	2	3.7
Stage		
I	3	5.6
II	5	9.3
III	9	16.7
IV	37	68.5
Tumor site		
Tongue	20	37
Tonsil	17	31.5
Larynx	10	18.5
Verbal base	3	5.6
Hypopharynx	2	3.7
Gl. submandibularis	1	1.9
Floor of mouth	1	1.9
HPV status		
HPV−	24	44.4
HPV+	20	37
Non-defined	10	18.5

The tumor tissues, metastatic and control lymph nodes were minced into small pieces with scissors and digested in RPMI 1640 containing 1 mg/mL of Collagenase D and 0.05 mg/mL of DNase I (both from Roche, 11088866001, 11284932001) for 30 min at 37°C under permanent gentle rocking motion. Afterwards, the specimens were passed through a 100-μm nylon cell strainer (BD Biosciences, 352360) and washed with PBS. The PBMCs were isolated from the peripheral blood by centrifugation on a Ficoll-Paque density gradient (GE Healthcare, 17-1440-03).

### Flow cytometry

Single cell suspensions from peripheral blood, tumor tissue and control lymph nodes were used for cell surface labeling using monoclonal antibodies (mAbs) against CD3, CD8^+^, CD11 c, CD14, CD16, CD19, CD20, CD45, CD45RA, CD45RO, CD56, CD62 L (Exbio), CD4^+^, CD123 (eBioscience, 12-1239-41), HLA-DR (BD Biosciences, 335830) and CCR7 (BioLegend, 353220) for detection of myeloid DCs (mDCs characterized as CD45+LIN-HLA-DR+CD14-CD11c+), plasmacytoid DCs (pDCs; CD45+LIN-HLA-DR+CD14-CD123+), monocytes/macrophages (M/Mϕ; CD45+LIN-HLA-DR+CD14+) and naïve T lymphocytes (defined as CD3+CD4+CD45RA+CD45RO-CCR7+CD62L+). The following anti-human mAbs were used for staining regulatory T cells (Tregs): anti-CD3 (Exbio, A7-202-T100), anti-CD4^+^ (eBioscience, 25-0049-42), anti-CD8^+^ (Exbio, 1 T-207-T100), anti-CD25 and anti-CD127 (BioLegend, 302626, 351318) for surface labeling, which was followed by fixation and permeabilization using the FoxP3 staining buffer set (eBioscience) and intracellular staining with anti-FoxP3 (eBioscience, 53-4776-42) and anti-Helios (BioLegend, 137216) antibodies. For analysis of Th17 and Th1 lymphocytes, cell suspensions were stimulated for 4 h with 50 ng/mL of PMA and 1 μg/mL of ionomycin (Sigma-Aldrich, P8139-1MG, I0634-1MG) in the presence of Brefeldin A (BioLegend, 420601) before intracellular staining. Next, the cells were stained with anti-CD3 (Exbio), anti-CD4^+^ (eBioscience), anti-CD8^+^ (Exbio), anti-PD-1 (BioLegend, 329908) and anti-Tim-3 (BioLegend, 345006) antibodies, fixed, permeabilized and labeled with mAbs against IL-17 (BioLegend, 512310) and IFNγ (BD Biosciences, 554551). The cells were analyzed on a BD FACSCanto II (BD Biosciences) and evaluated with FlowJo software (TreeStar).

### Chemokine and cytokine analysis

For the analysis of IL-1β, IL-2, IL-4, IL-6, IL-10, IL-12, IL-17, IL-21, IL-23, TNFα, IFNγ, CCL5, CCL17, CCL19, CCL20, CCL21, CCL22, CXCL9, CXCL10, and CXCL12 in culture supernatants harvested from tumor tissue-derived cell suspensions, a Quantibody Array Kit (Raybiotech) was used. Cell suspensions at the concentration of 1 × 10^6^/mL in the presence or absence of PMA and ionomycin were cultured for 24 h in RPMI 1640 supplemented with 10% FCS, Glutamax and penicillin-streptomycin (Invitrogen, A12860-01, 15140-130). The supernatants were then collected and stored at -80°C until use.

### RNA extraction and quantitative real time PCR

Total RNA was extracted from 2 × 10^6^ tumor-tissue derived cells using an RNA Easy Mini Kit (Qiagen). RNA concentrations were determined with a NanoDrop© 2000 c UV-Vis spectrophotometer (Thermo Scientific), and the RNA integrity was assessed using an Experion automated system (BioRad). cDNA was synthesized from total RNA using the M-MLV reverse transcriptase (Invitrogen) and amplified by quantitative real time PCR in the presence of primers and TaqMan© probes specific for *Cox-2*, *PD-1*, *PD-L1* and *Tim-3*, as well as the β-actin housekeeping gene, which was used as an internal reference. All primers and probes were commercially synthesized (TIB MOLBIOL). The identity of the qPCR products in each assay was verified by sequencing. The relative expression of the target genes was normalized to the expression of β-actin.

## HPV Detection

### Tumor samples

The pathologist obtained two side-by-side sections of the tumor from the primary site. One of the paired sections from each anatomical location was labeled, snaps frozen in liquid nitrogen, and stored for future analysis. The other section from each pair was fixed in 10% neutral formalin and paraffin embedded. From each paraffin block, the first and last sections were histologically analyzed to confirm that the sections in between that were designated for the detection of viral nucleic acids and immuno-histochemical (IHC) analysis contained at least 10% of the tumor cells from the entire volume of the sample. Both DNA and RNA, were extracted from the tumor tissue using the Ambion Recover All TM Total Nucleic Acid Isolation Kit for FFPE Tissues (Applied Bioscience) as previously reported.^49^ Care was taken to avoid sample cross-contamination.

### PCR

All procedures have been described in detail previously.^49,50^ HPV DNA detection was performed by PCR with primers specific for the L1 region (GP5^+^/GP6^+^) as described previously.^51^ As an internal control, a 110-bp fragment of the human β-globin gene was amplified.^52^ HPV typing was performed by reverse line blot hybridization (RLB) with probes specific for 37 types as specified in detail by van den Brule et al.^53^ From the total RNA, cDNA was prepared by reverse transcription. The absence of contaminating DNA was confirmed by amplification of the internal GAPDH internal control gene.^54^ As a control for the integrity of the mRNA, the β-globin gene was amplified. Amplification of the HPV 16E6*I mRNA oncoprotein was performed with primers that amplify the 86-bp fragment.^55^

### Immunohistochemical analysis

IHC examination was performed as described previously.^49^ Briefly, the antibody *p*16INK4 a (purified mouse anti-human p16, Clone G175-405, BD PharMingen TM, dilution 1:100) was used. The intensity of staining (graded + to +++) and the proportion of cells stained (scored in percentages) were evaluated. For *p*16 immunostaining, the location of the signal (cytoplasmic and/or nuclear) was also specified. A semi-quantitative evaluation was performed. The samples considered positive for *p*16 expression exhibited more than 50% positive cells and nuclear and/or cytoplasmic staining.

### Statistical analysis

Statistical analyses were performed using Statistica® 10.0 software (StatSoft).

The parametric assumptions of the data were verified using the Kolmogorov–Smirnov test for normality. The homogeneity of the variances was tested by the Levene test. The differences between HPV-positive and HPV-negative tumor samples were analyzed using a general linear model (GLM), and age was added as a covariate. The differences between control lymph nodes and tumor tissue were analyzed using a paired t-test. The results were considered statistically significant when *p* < 0.05.
